# Prevalence and clinicopathological characteristics of de novo metastatic cancer at a major radiotherapy centre in West Africa: a cross-sectional study

**DOI:** 10.3332/ecancer.2024.1805

**Published:** 2024-11-29

**Authors:** Joseph Daniels, Onesmus Iinekela Amunyela, Andrew Yaw Nyantakyi, Edwina Ayaaba Ayabilah, Judith Naa Odey Tackie, Kofi Adesi Kyei

**Affiliations:** 1 National Centre for Radiotherapy, Oncology and Nuclear Medicine, Korle Bu Teaching Hospital, Accra, Ghana; 2 Department of Radiography, University of Ghana, Legon, Accra, Ghana; a https://orcid.org/0000-0002-1466-150X; b https://orcid.org/0009-0005-3479-7219; c https://orcid.org/0000-0003-0742-6007; d https://orcid.org/0009-0009-8399-4920; e https://orcid.org/0000-0002-2703-5508; f https://orcid.org/0000-0003-3485-5368

**Keywords:** cancer prevalence, de novo metastasis, treatment naïve patients, cancer treatment, metastatic adenocarcinoma, radiotherapy

## Abstract

**Background:**

Cancer is a major public health challenge in West Africa, with a significant proportion of cancer-related deaths attributed to distant metastasis. De novo metastatic cancer (DnMC), where metastasis is detected at diagnosis, presents considerable therapeutic challenges, particularly in limited-resource settings where novel treatments are often unavailable and/or unaffordable.

**Aim:**

To determine the prevalence, incidence and clinicopathological characteristics of patients diagnosed with DnMC at a major radiotherapy center in West Africa.

**Methods:**

This was a single-institution-based quantitative cross-sectional study. Data on the prevalence and incidence of DnMC were retrieved from a hospital-based cancer registry whereas patients’ demographic and clinicopathologic data were extracted from patients’ medical records and analysed with STATA software (version 16). Descriptive statistics were used to summarise patient- and tumour-related characteristics.

**Results:**

The prevalence and incidence of DnMC were 15.2% and 5.3%, respectively, with a 36% overall incidence rate of metastatic cancer. The mean age was 50.9 years (SD 15.2), ranging from 15 to 90 years, with a male-to-female ratio of 1:1.6. Also, 28.8% had a history of alcohol intake whereas 13.7% were (tobacco) smokers. Additionally, 10.3% of the patients had a positive family history of cancer. Pain (28.2%) was the most common presenting symptom, followed by bleeding (16.5%). In all, 34.9% had an Eastern Cooperative Oncology Group (ECOG) performance status of 0 whereas 33.3% were ECOG 1. The gastrointestinal tract (25.8%) and breasts (17.6%) were the most frequent primary cancer sites, whereas 4.4% had metastatic cancers of unknown primary origin. The most frequent sites of distant metastasis were the lungs (34.6%), liver (28.9%) and bone (13.8%). Adenocarcinoma was the most prevalent histological type (35.2%).

**Conclusion:**

There was a relatively high rate of DnMC compared with high-income countries, emphasising the need for early detection and expanded access to comprehensive cancer care in limited-resource settings.

## Introduction

### Background

Cancer poses a significant public health challenge in West Africa, even as the number of diagnosed cases continues to rise with 19.3 million new cases diagnosed worldwide in 2020 alone [1]. Despite groundbreaking advancements in screening, early detection and treatment, approximately two-thirds of cancer-related deaths particularly among patients with solid tumours, can be attributed to distant metastasis [2]. Different types of cancer exhibit varying tendencies to spread to various parts of the body. For instance, breast cancer is likely to metastasise to the bone, brain, liver or lungs, whereas colorectal cancer tends to spread to the liver, lungs or peritoneum [3]. Metastatic cancer can present as a recurrent disease after previous treatment or as a progressive disease during ongoing but ineffective treatment [4].

De novo metastatic cancer (DnMC), however, refers to the distant spread of cancer detected at the time of initial diagnosis before the commencement of any antineoplastic therapy. The occurrence of DnMC is often associated with late diagnosis of the primary tumour, aggressive tumour biology, lack of efficient screening tools and limited access to appropriate diagnostic investigations [5, 6]. Treatment options for DnMC generally comprise radiotherapy, chemotherapy, immunotherapy as well as targeted and hormonal therapy. Although surgery has limited use in the management of metastatic cancer, it may still be employed to remove isolated metastatic lesions, particularly in patients with a low burden of metastatic disease, controlled primary tumour and a good overall performance status. Surgery may also be used for palliative purposes such as pain relief, bleeding control or alleviating obstruction caused by metastatic or locoregional tumours. Although DnMC is treatable, it tends to be incurable and is associated with an inferior prognosis. DnMC presents a significant therapeutic challenge, especially in low-middle-income-countries (LMICs) where a lot of novel therapeutics are unaffordable and/or unavailable [7].

Despite high rates of distant metastatic disease and cancer-related deaths in Sub-Saharan Africa, there is little published information on the prevalence of DnMC in this region. There is also a paucity of reliable data on the scope and magnitude of the challenge of DnMC in the Ghanaian context. Gaining insight into the clinicopathological characteristics of patients in West Africa with DnMC can assist healthcare providers in developing tailored management strategies. Healthcare providers can also optimise the management of DnMC by implementing targeted interventions to promote early diagnosis and treatment thus enhancing overall patient care.

### Aim

The aim of the study was to determine the prevalence, incidence and clinicopathological characteristics of patients with DnMC at one of the major radiotherapy centers in the West African subregion.

## Methods

### Study design and setting

This research was a single-institution-based quantitative cross-sectional study conducted at a major radiotherapy and oncology centre in Accra, Ghana. The study site is a prominent public cancer treatment facility that offers comprehensive care to cancer patients in Ghana and the West African subregion. The centre also hosts one of the largest cancer registries in Ghana that maintains data on cancer cases managed at the third-largest hospital in Africa.

### Participants

The study involved metastatic cancer patients treated at the study site between 01 January 2021 and 31 December 2022. For the determination of the prevalence of DnMC, total population sampling was used to identify all patients ≥15 years old diagnosed with metastatic cancer of any primary site at the time of presentation, who were confirmed to be cancer-treatment naïve. A purposive sampling technique was subsequently used to recruit consenting eligible patients with DnMC for clinicopathological profiling. Patients with confirmed metastatic cancers of unknown primary (CUP) origin were also included in the study. Histopathological and radiological investigations such as computed tomography (CT), mammography, Technetium-99m scintigraphy and X-ray imaging as well as magnetic resonance imaging (MRI) and ultrasonography, were used to confirm both primary and metastatic cancers sites. Metastatic patients with unavailable or unreliable histopathological and/or radiological evidence of either primary and/or metastatic cancer were excluded. Metastatic cancer patients reporting for the first time at the study site with a previous history of cancer treatment were also excluded whether they had completed full treatment or not. Additionally, patients with incomplete medical records or unclear history of antineoplastic therapy were also excluded.

### Variables

- ‘Cancer of unknown primary’ was defined as the presence of distant metastasis at the time of initial diagnosis without any evidence of a primary cancer site despite a comprehensive diagnostic workup.
- Distant metastasis was defined as the spread of a primary tumour or cancer of unknown primary origin to distant sites of the body.
- ‘DnMC’ was defined as the distant spread of cancer detected at the time of initial diagnosis prior to the commencement of any antineoplastic therapy.
- Prevalence of DnMC was defined as the ratio of the total number of patients with confirmed DnMC (267 patients) to the total number of patients with confirmed metastatic disease (1,759 patients).
-
$$\mathrm{Pr}\mathrm{evalence}\;\mathrm{of}\;\mathrm{DnMC}\;=\;\frac{\mathrm{Total}\;\mathrm{number}\;\mathrm{of}\;\mathrm{patients}\;\mathrm{diagnosed}\;\mathrm{with}\;\mathrm{DnMC}}{\mathrm{Total}\;\mathrm{number}\;\mathrm{of}\;\mathrm{patients}\;\mathrm{with}\;\mathrm{distant}\;\mathrm{metastasis}\;\mathrm{at}\;\mathrm{pesentation}}\;\times \;100\%$$
-
$$\mathrm{Incidence}\;\mathrm{of}\;\mathrm{DnMC}\;=\;\frac{\mathrm{Number}\;\mathrm{of}\;\mathrm{new}\;\mathrm{cancer}\;\mathrm{patients}\;\mathrm{diagnosed}\;\mathrm{with}\;\mathrm{DnMC}}{\mathrm{Total}\;\mathrm{number}\;\mathrm{of}\;\mathrm{new}\;\mathrm{cancer}\;\mathrm{patients}}\;\times \;100\%$$
-
$$\mathrm{Incidence}\;\mathrm{of}\;\mathrm{metastatic}\;\mathrm{cancer}\;=\;\frac{\mathrm{Number}\;\mathrm{of}\;\mathrm{new}\;\mathrm{cancer}\;\mathrm{patients}\;\mathrm{with}\;\mathrm{distant}\;\mathrm{metastasis}}{\mathrm{Total}\;\mathrm{number}\;\mathrm{of}\;\mathrm{new}\;\mathrm{cancer}\;\mathrm{patients}}\;\times \;100\%$$
- Total number of patients diagnosed with DnMC at presentation = 267
- Number of new cancer patients diagnosed with distant metastasis at presentation = 1,759
- Total number of new cancer patients seen during the study period = 4,879.

### Data sources

Secondary data regarding the total number of patients diagnosed with DnMC, the overall number of cancer patients who presented with metastatic disease and the total number of new cancer cases managed at the study site between 2021 and 2022 were obtained from the hospital-based cancer registry. Data on the demographic and clinicopathological characteristics of the study participants were extracted from hospital-based medical records into a Microsoft Excel worksheet. The parameters recorded included patients’ age, sex, body mass index (BMI), performance status, clinical symptoms, tumour histology and primary as well as metastatic cancer sites. The treatment modalities used in the management of the patients were also recorded as well as the intention of treatment.

### Bias

To reduce the tendency of inadvertently excluding eligible patients, screening of patients for the study was done by two independent reviewers. The outcome of their work was compared for consistency and in case of any discrepancies, a third reviewer stepped in to review the records of the patient(s) in question. To eliminate potential selection bias due to the consent process and patient mortality before recruitment, the prevalence rate of DnMC was determined based on total population sampling of all recorded cases of DnMC at the study site during the study period.

### Study size

The total number of new cancer patients who presented for treatment at the study site during the study period was 4,879, among whom 1,759 had distant metastatic disease. A total of 267 of these patients had DnMC at presentation (based on data from the hospital-based cancer registry). Purposive sampling was used to select 159 patients with confirmed DnMC for clinicopathological profiling. All 1,759 patients who presented with metastatic cancer were screened for eligibility, out of which 177 patients with DnMC were found to be eligible based on the inclusion and exclusion criteria. Eighteen patients were excluded due to incomplete medical records, unavailable histopathological evidence of primary tumour or unresolved uncertainty concerning antineoplastic treatment received by the patients prior to the detection of distant metastasis.

### Statistical methods

The clinicopathological data of 159 purposively selected patients who provided written informed consent were analysed, using STATA software (version 16). Descriptive statistics were used to summarise patient- and tumour-related characteristics. Frequency distributions such as percentages and means with standard deviations were used to summarise the data. The issue of missing data was addressed by imputing results for missing variables.

### Ethical considerations

Ethical approval was obtained from the institutional review board of the School of Biomedical and Allied Health Sciences of the University of Ghana, Legon prior to the commencement of this research (reference number: SBAHS/AA/RAD/10943323/2022-2023). Throughout the study, patient confidentiality was strictly maintained and the data was discretely handled. Informed consent was obtained from participants, and to ensure patient privacy, all patient-identifying information was removed prior to data analysis. For patients between 15 and 17 years, written informed consent was obtained either from their parents or a legally recognised guardian (or legally authorised representative).

## Results

### Prevalence and incidence of DnMC and metastatic cancer

The prevalence and incidence rates of DnMC were 15.2% and 5.3%, respectively, whereas the overall incidence rate of distant metastatic cancer was 36%.

### Baseline characteristics

**Figure 1.**
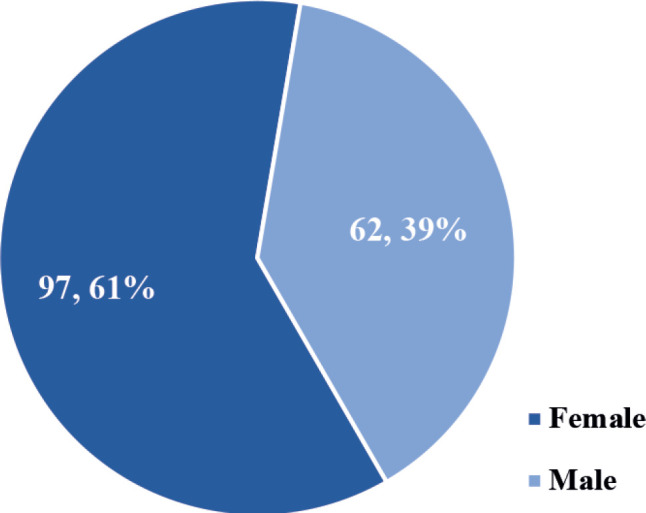
Gender distribution of the study participants (*N* = 159).

The study involved a total of 159 patients with confirmed DnMC. The participants were predominantly female (*n* = 97, 61%) as depicted in Figure 1.

**Figure 2.**
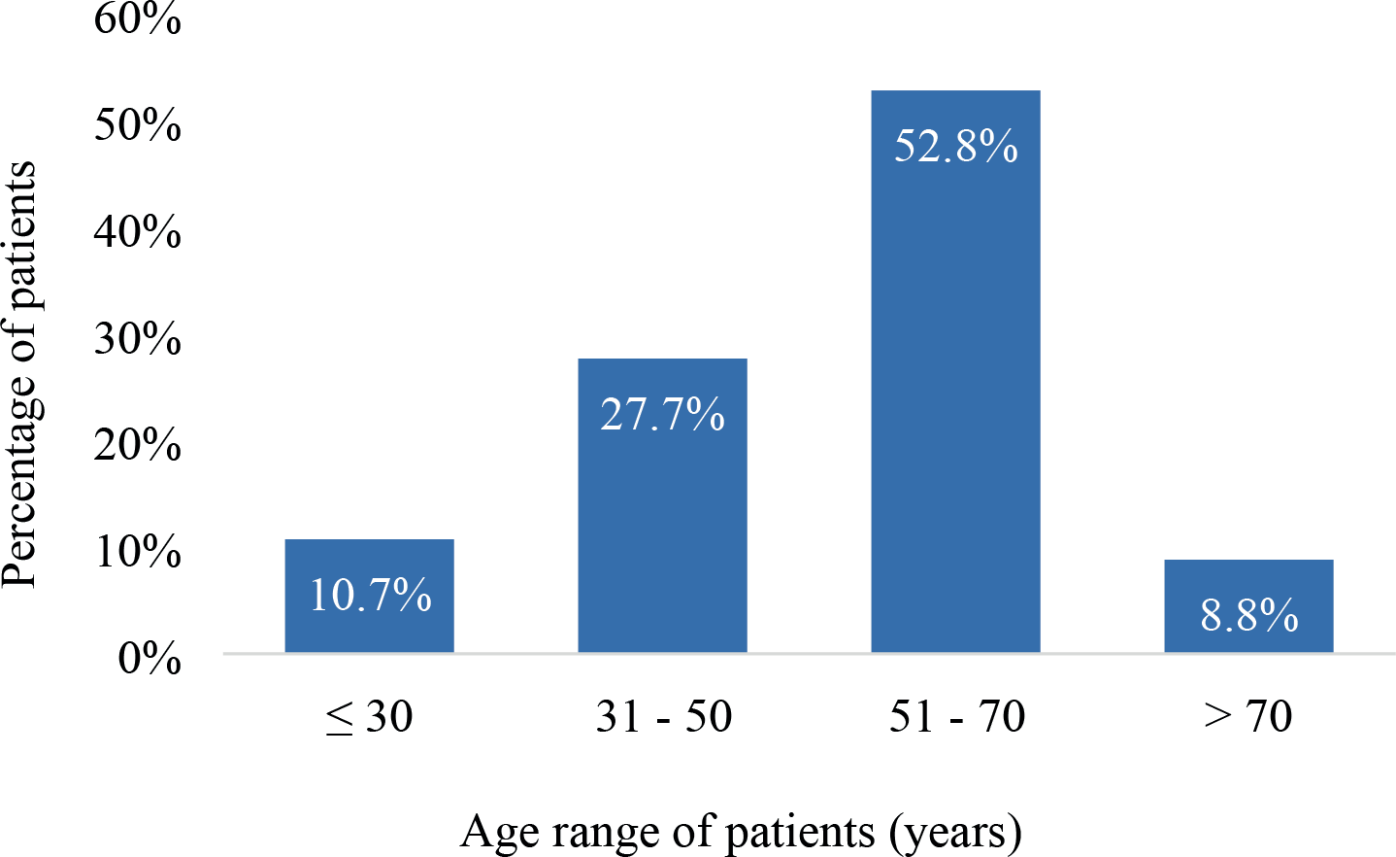
Age distribution of the study participants (*N* = 159).

The mean age was 50.9 years (SD 15.2) ranging from 15 to 90 years. A considerable proportion (52.8%) were between 51 and 70 years old. In all, 10.7% were ≤30 years old whereas 8.8% were older than 70 years as illustrated in Figure 2.

Table 1 summarises the distribution of BMI categories among the participants. In all, 82 patients (51.6%), were within the healthy BMI range of 18.5 to 24.9 kg/m² whereas 18 (11.2%) were in the obesity category. Also, 33 patients (20.8%) were underweight whereas 26 (16.4%) were overweight.

### Risk factors and comorbidities

**Figure 3.**
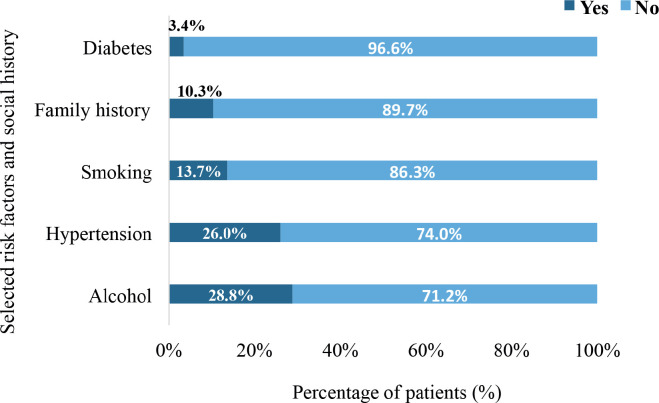
The distribution of comorbidities and risk factors among the participants (*N* = 159). Diabetes refers to diabetes mellitus. Smoking specifically refers to smoking of tobacco whereas hypertension refers to hypertensive heart disease.

In all, 28.8% had a history of alcohol intake whereas 13.7% had a history of tobacco smoking. Also, 26% were simultaneously diagnosed with hypertensive heart disease whereas 3.4% had diabetes mellitus. In all, 10.3% of the patients had a positive family history of cancer (Figure 3).

### Clinical signs and symptoms

**Figure 4.**
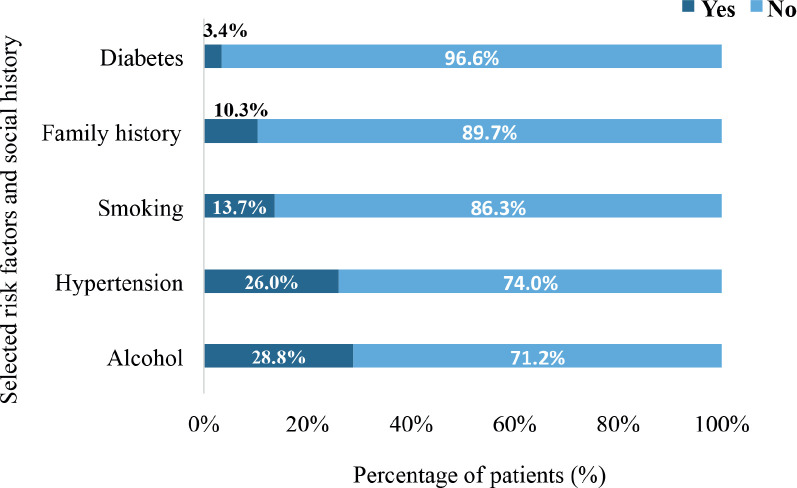
Signs and symptoms experienced by patients at the time of initial diagnosis. LUTS: lower urinary tract symptoms. Pain symptoms of patients encompassed pain in different body parts such as the abdomen, neck and limbs. Bleeding refers to either bleeding ‘per vaginum’ or per rectum. Breast symptoms included nipple retraction, discharge, ‘peau d‘orange’, breast ulceration and the presence of a lump in the breast. Swellings comprised abnormal masses in the axilla, on extremities or other body parts. Bowel symptoms were mainly diarrhea and constipation. Cachexia represents patients who experienced unintentional weight loss >10% of their baseline within six months preceding their diagnosis. Other symptoms were dysphonia, visual impairment, nausea and vomiting.

Figure 4 demonstrates the spectrum of clinical signs and symptoms experienced by the patients between the time of onset of cancer and the time of their histologically confirmed diagnosis. The signs and symptoms experienced by the patients were not mutually exclusive. Some patients experienced multiple signs and/or symptoms whereas others experienced only one sign or symptom. Overall, pain (28.2%) was the most frequently reported symptom followed by bleeding (16.5%) and breast symptoms (14.1%). Also, 3.5% of the patients reported headache, cough, cachexia and bowel symptoms.

### Performance status

**Figure 5.**
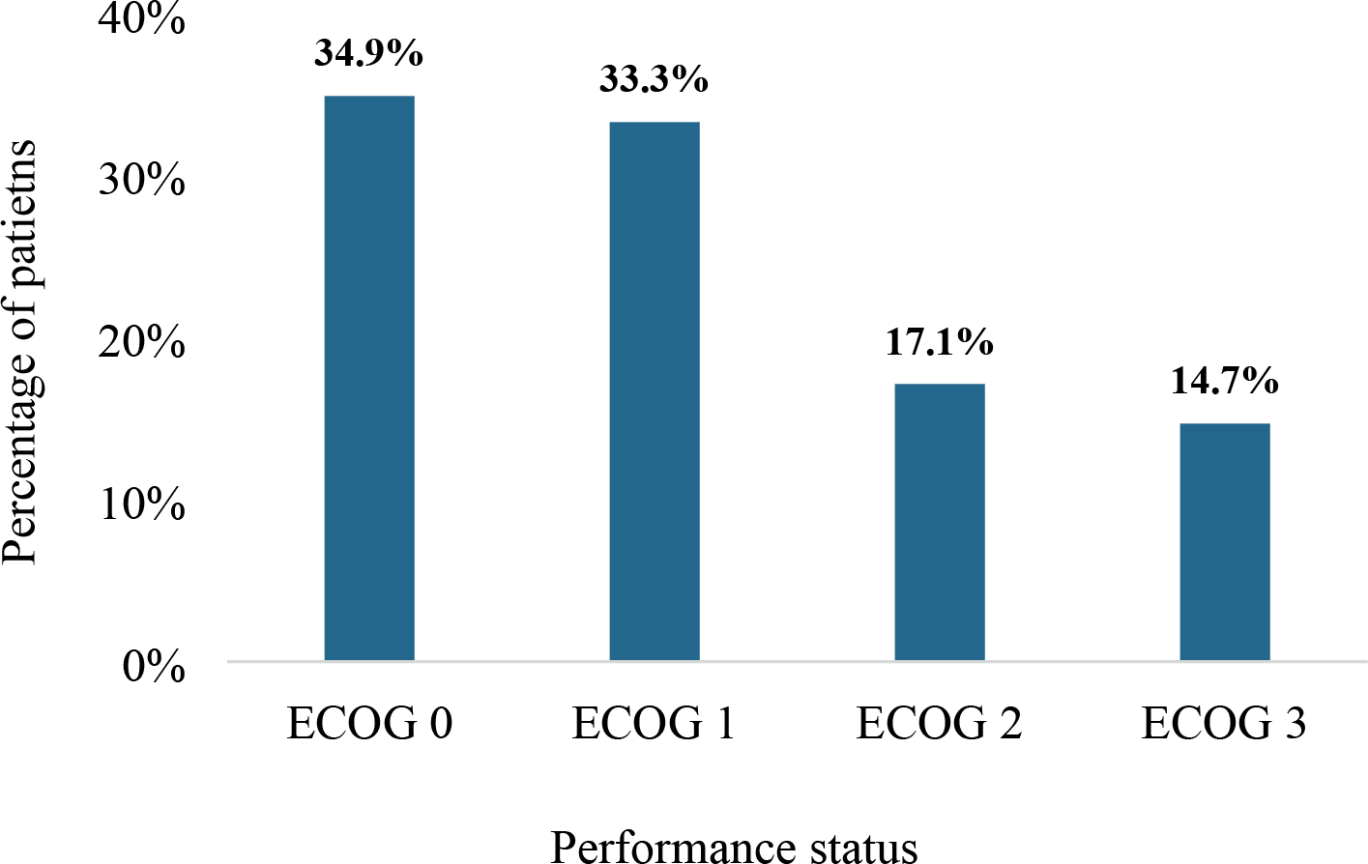
The performance status of patients with DnMC. ECOG: Eastern Cooperative Oncology Group. The scale summarises the functional status of a cancer patient based on their ability to care for themselves, and undertake physical activity and activities of daily living [8, 9]. ECOG 0 implies intact ability for pre-disease functioning without any restriction whereas ECOG 1 refers to a restriction in strenuous physical ability with preserved ability to undertake normal work activity. ECOG 2 refers to ambulatory patients who are capable of selfcare and stay up more than 50% of their waking hours. ECOG 3 refers to patients capable only of limited selfcare and are confined to a bed of chair >50% of their waking hours.

Figure 5 illustrates the Eastern Cooperative Oncology Group (ECOG) performance status of the study participants. In all, 34.9% had a performance status of ECOG 0 whereas 33.3% were ECOG 1. Also, 17.1% and 14.7% had performance statuses of ECOG 2 and 3, respectively. There were no patients who had a performance status of either ECOG 4 or 5.

### Detection of sites of distant metastasis

In the detection of metastatic cancer sites among the participants, ultrasonography was the most frequently used imaging modality (60.3%), followed by X-rays (45.2%) and CT scans (32.7%) as summarised in Table 2. MRI and bone scintigraphy were less commonly used, 9.4% and 8.2%, respectively.

### Primary cancer sites

**Figure 6.**
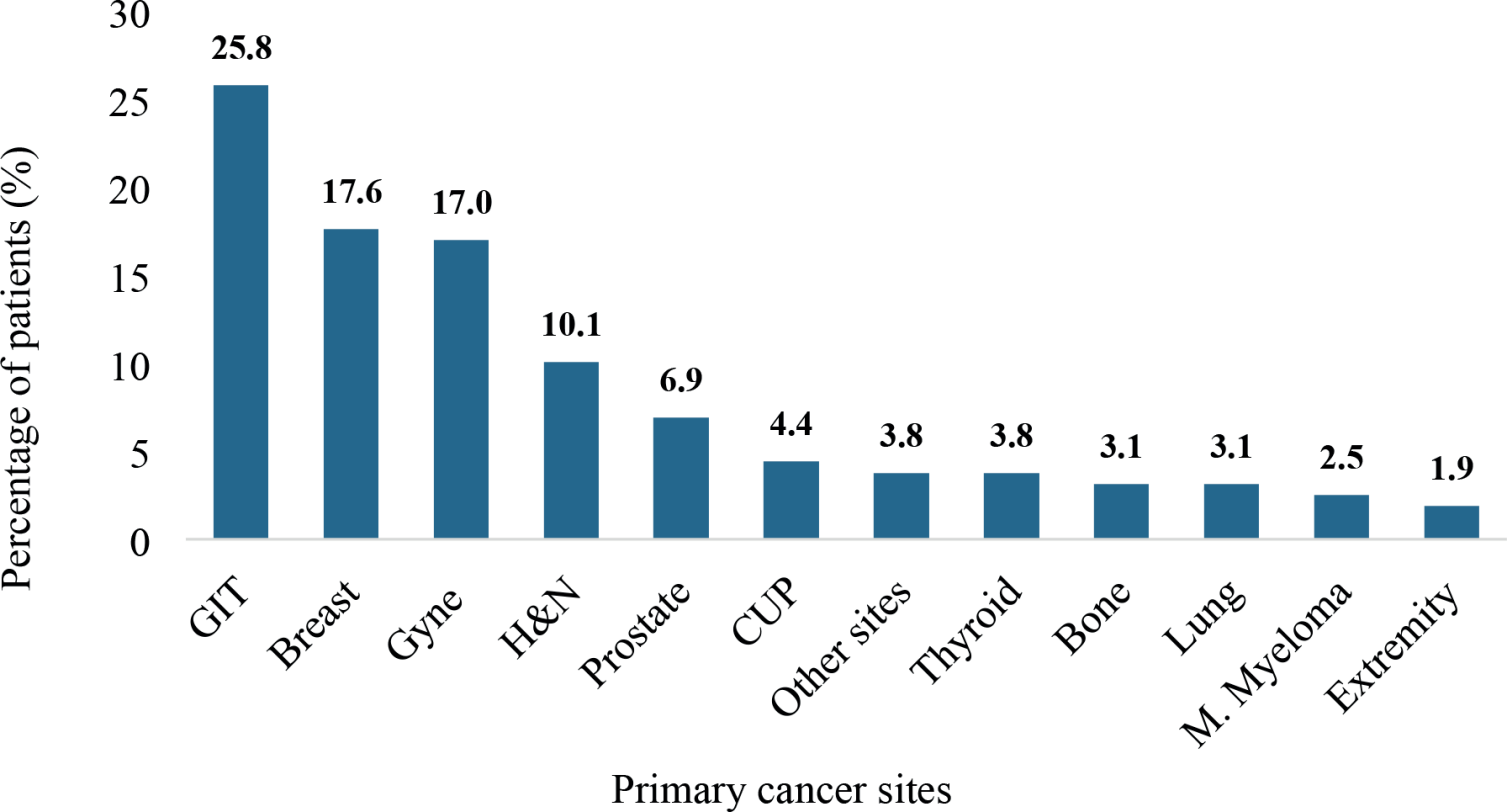
The primary cancer sites of patients with de novo metastasis. GIT: gastrointestinal tract, Gyne: gynecological system, H&N: head and neck, CUP: cancer of unknown primary, M. myeloma: multiple myeloma. Cancers diagnosed in the GIT were colorectal, anal, gastric, pancreatic and esophageal cancers as well as hepatobiliary cancers. Breast cancers are essentially invasive ductal carcinomas. The primary tumours of the gynecological system comprised cervical, ovarian, endometrial and vulva cancers. Primary cancers diagnosed in the H&N region included nasopharyngeal, hypopharyngeal, laryngeal oropharyngeal, maxillary sinus, parotid and other salivary gland tumours. Cancers of the extremities comprised sarcomas of either upper or lower limbs. Other primary cancer sites not otherwise represented in figure 8 were tumours of the skin, kidney, hematological and soft tissue sarcomas of the back.

De novo metastases were associated with different primary cancer sites among 95.6% of the patients. However, 4.4% had metastatic CUP sites. The gastrointestinal tract (25.8%) was the commonest primary site followed by the breast (17.6%), gynecological system (17.0%), the head and neck region (H&N) (10.1%) and prostate (6.9%) in order of decreasing proportion as illustrated in Figure 6.

### Sites of distant metastasis

**Figure 7.**
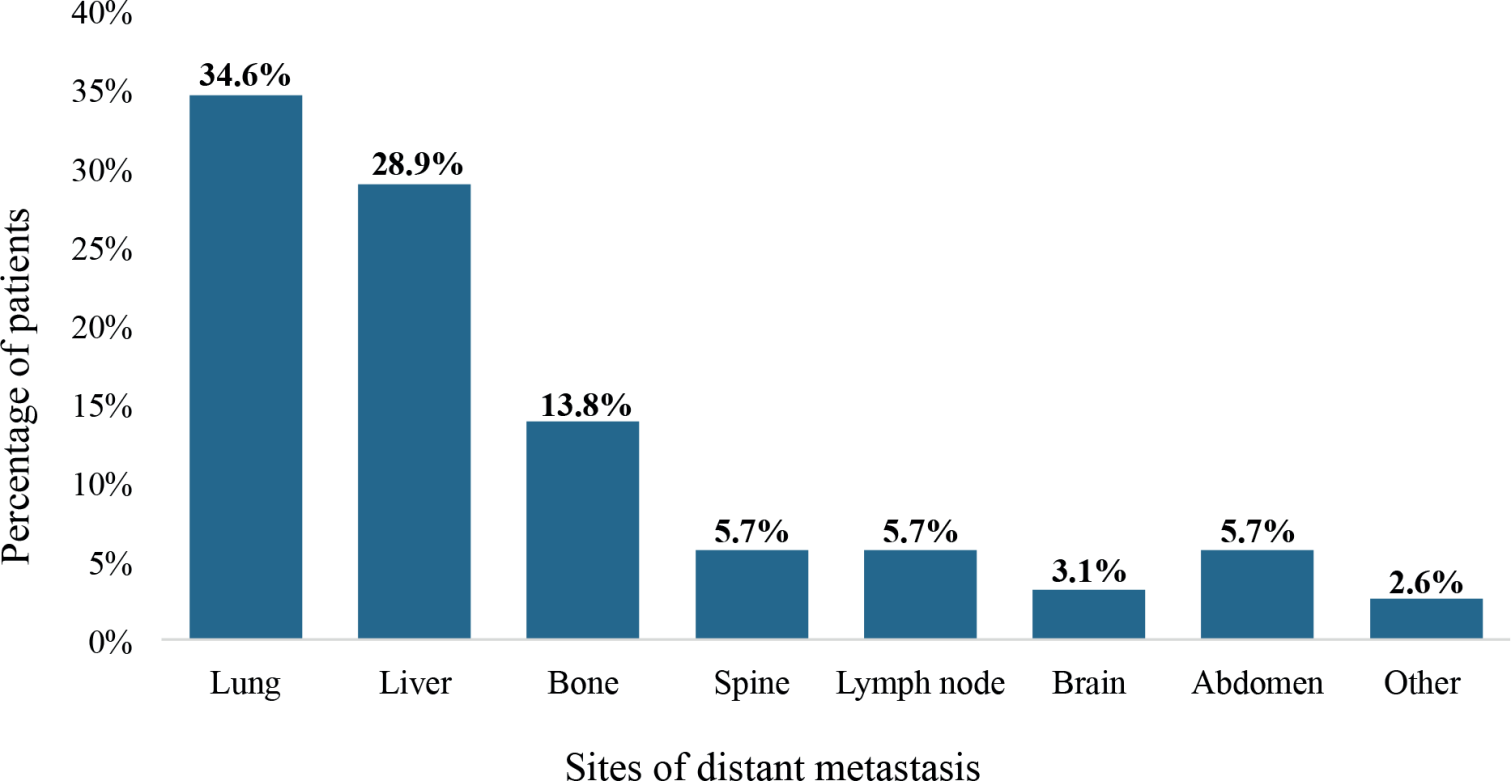
The metastatic sites of patients with de novo metastasis. Metastasis in the abdomen comprised distant spread to the mesentery, peritoneum and intraabdominal viscera besides the liver. ‘Other’ metastatic sites included the kidneys, spleen and adrenal glands.

The lungs were the most frequent sites of distant metastasis among the patients (34.6%), followed by the liver (28.9%) and bone (13.8%). Also, spine, non-regional lymph node and brain metastases were detected among 5.7%, 5.7% and 3.1% of the patients, respectively, as illustrated in Figure 7.

### Histological types

**Figure 8.**
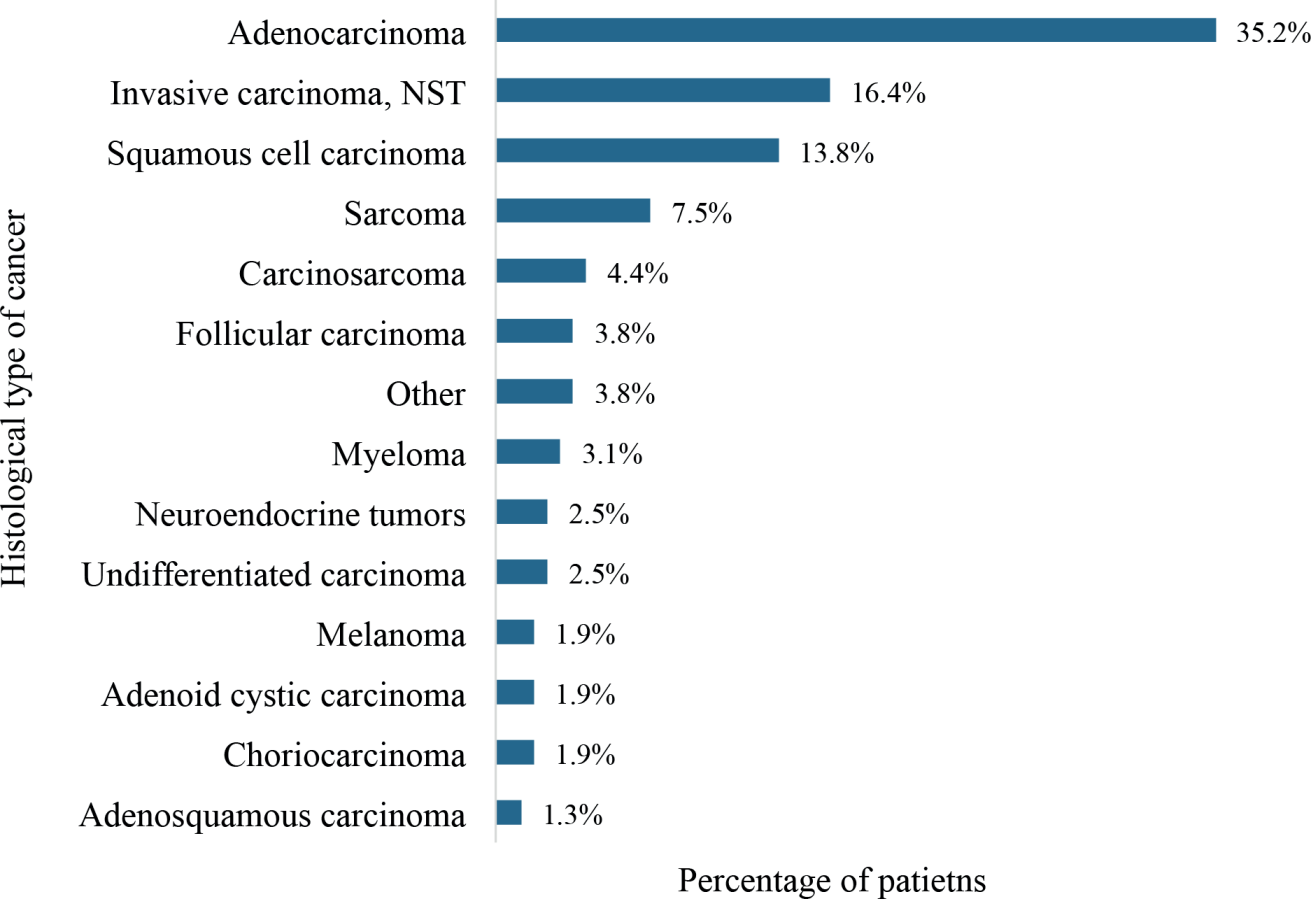
Histological types of cancers with de novo metastasis. NST: no special type. Histological cancer types that were recorded in only a single patient were grouped together as ‘other’. This group comprised medullary carcinoma, mucinous carcinoma, papillary carcinoma, transitional cell carcinoma, yolk sac tumour and retinoblastoma.

The commonest histological type was adenocarcinoma (35.2%) followed by invasive carcinoma of no special type (16.4%), squamous cell carcinoma (13.8%) and sarcoma (7.5%) as shown in Figure 8. Also, 7.5% were soft tissue sarcomas of various body sites whereas 3.8% were follicular carcinomas of the thyroid gland. Carcinosarcomas of the uterine corpus accounted for 4.4% of the cases.

### Treatment modalities

**Figure 9.**
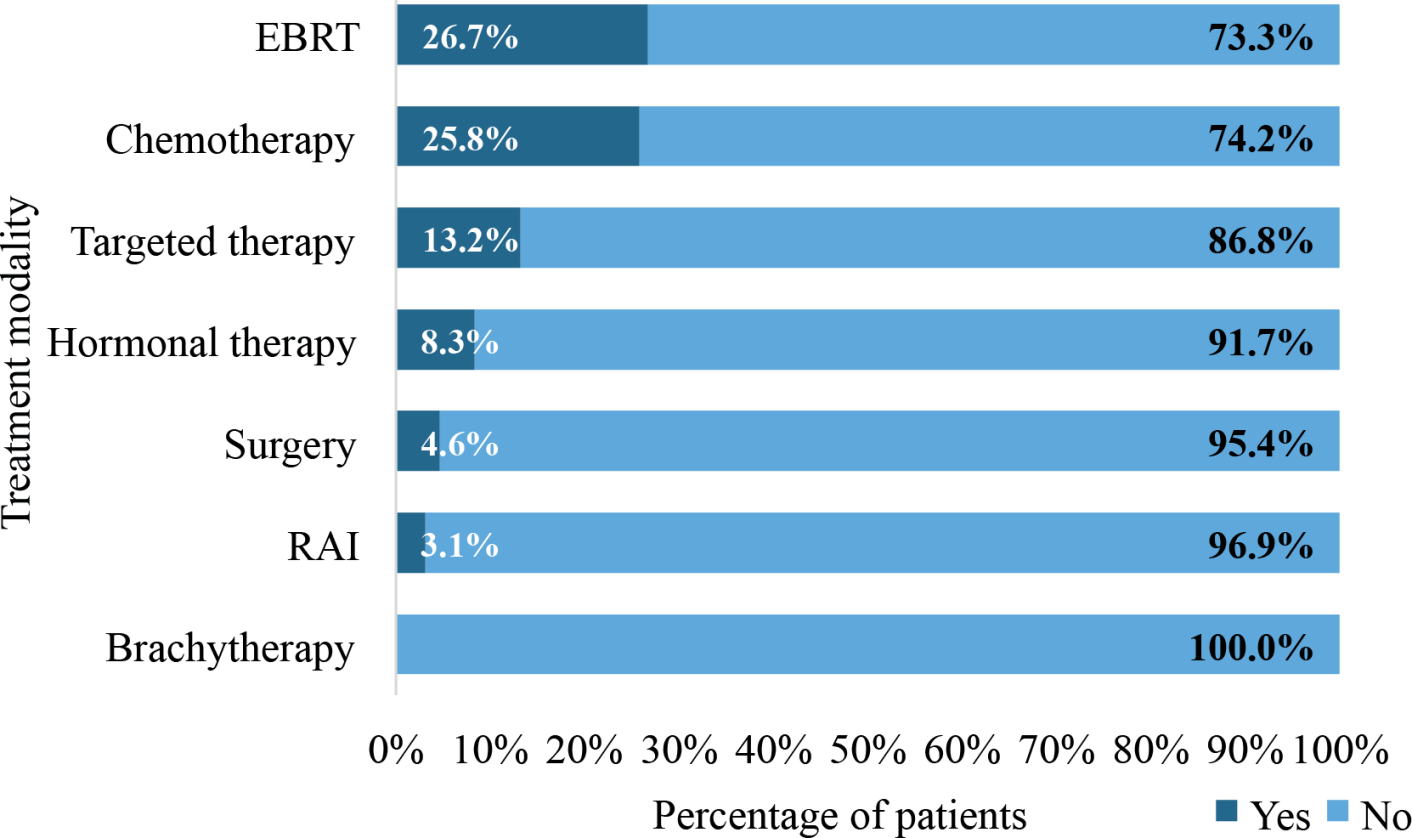
Treatment modalities utilised in managing patients with de novo metastasis. EBRT: external beam radiotherapy, RAI: radioactive iodine therapy.

Figure 9 illustrates the pattern of treatment modalities utilised in the management of patients with DnMC. The most frequently utilised treatment modality was external beam radiotherapy (EBRT), which was administered to 58 patients (26.7%). In all, 56 patients (25.8%) received chemotherapy whereas 18 patients (8.3%) received hormonal therapy. Only, 10 patients (4.6%) underwent surgery. Radioactive iodine therapy was administered to 3.1% of the patients whereas 13.2% received targeted therapy. None of the patients 7was treated with brachytherapy.

### Intention of treatment

Table 3 illustrates the distribution of treatment intention among the patients. In all, 138 (86.7%) received palliative treatment whereas 8 patients (5%), were treated with curative intent. Additionally, 13 patients (8.3%) received best supportive care alone.

## Discussion

The study found DnMC prevalence and incidence rates of 15.2% and 5.3%, respectively, with a 36% overall incidence rate of distant metastatic cancer, indicating a substantial burden of metastatic disease at the time of cancer diagnosis in this population. In high-income countries such as the United States of America and many European countries, the prevalence of DnMC is considerably lower, often in the range of 5%–10% depending on the cancer type [10–12]. Similarly, a low DnMC prevalence rate of 9.5% has been reported in other developed nations such as Canada and Australia [13]. In sharp contrast, disturbingly high prevalence rates have been reported in several LMICs. Agboola [14] documented a DnMC prevalence rate of approximately 18% in West Africa whereas Akinremi [15] reported a prevalence rate exceeding 20% in sub-Saharan Africa. In Kenya, Mburu [16] reported a prevalence rate of 17%, which is closely aligned with the data from Ghana reported in this study. Some studies have also reported that more than 20%–30% of cancer patients in Nigeria and Kenya present with DnMC [17, 18]. However, Onyema [19] documented a slightly lower prevalence of approximately 14% in Nigeria. The broad spectrum of reported DnMC prevalence rates suggests potential regional disparities within sub-Saharan Africa. Interestingly, Mohan [20] also reported a lower prevalence rate of 12% in India, highlighting significant variability even among LMICs .

The disparity in the prevalence of DnMC between high- and low-income countries may be intricately linked with disparities in healthcare access, utilization and the quality of care received by cancer patients. High-income countries generally have more advanced healthcare infrastructure, comprehensive cancer screening programs, and higher public health awareness, which facilitate earlier cancer detection and reduce the likelihood of metastatic diagnoses [11, 21]. In contrast, low-income countries, such as Ghana, often struggle with limited healthcare access, fewer screening initiatives and lower cancer awareness, leading to more cancers being diagnosed at an advanced stage [17]. Patients from limited-resource settings and marginalised populations are disproportionately affected by DnMC due to socioeconomic factors such as income levels, education, insurance status and geographic location which influence cancer screening rates, timely diagnosis and access to advanced cancer treatment options [22]. The prevalence and epidemiology of DnMC have significant implications for clinical practice, guiding strategies for risk assessment, screening, diagnosis, treatment selection and supportive care.

The study period (January 2021–December 2022) coincided with the COVID-19 pandemic, when healthcare systems globally, including Ghana, faced significant challenges such as restricted access to healthcare, delays in diagnosis, disruptions in cancer treatment and altered patient behaviours due to lockdowns, fear of infection and financial constraints. Although the study did not specifically assess the impact of COVID-19 on the care-seeking behaviour of cancer patients or delays in diagnosis, it is possible that these factors may have influenced the reported rate of DnMC. Additionally, pandemic-related disruptions in screening and referral pathways could have also resulted in fewer early-stage cancer diagnoses and increased presentation with advanced/metastatic disease. Nevertheless, the incidence and prevalence of DnMC found in this study underscore the need for multidisciplinary approaches to cancer care, especially in limited-resource settings.

Understanding the gender and age distribution of patients with DnMC is crucial for developing targeted screening programs and treatment strategies. Previous studies have demonstrated variations in the gender distribution of DnMC across different cancer types [23]. For example, de novo metastatic lung cancer tends to be more prevalent in men than women [24]. The high proportion of females with DnMC (61%) is consistent with the relatively higher incidence of cancer among females in Ghana and the fact that 3 of the top 6 most frequently diagnosed cancers in Ghana (i.e., cancers of the breast, cervix and ovary) primarily occur in females [25]. The observed gender distribution is not only consistent with known global patterns in cancer epidemiology but also underscores some unique aspects pertinent to the West African population, particularly regarding healthcare access, and socioeconomic factors that influence patient demographics [17, 26]. The gender disparity among patients with DnMC highlights a critical area of concern in terms of delayed diagnosis, low uptake of cancer screening and other factors that may disproportionately hinder the early diagnosis and treatment of cancer in women in the region. Conversely, the relatively lower proportion of male patients diagnosed with DnMC (39%) may reflect gender-specific health disparities in the utilization of healthcare services. Men may be less likely to seek medical care until symptoms are severe or until the disease has progressed to an advanced stage. In West Africa, cultural attitudes toward masculinity, combined with barriers such as financial constraints and limited access to healthcare facilities, contribute to delayed presentation and diagnosis of cancers in men [27]. This underscores the need for public health interventions targeting men, aimed at improving early detection and timely access to comprehensive cancer care.

The mean age of 50.9 years (SD 15.2) is consistent with the typical age range for cancer diagnosis globally. In particular, breast, prostate and gastrointestinal cancers tend to have an onset in middle-aged to older adults, with metastatic cancer diagnosis commonly occurring in the fifth and sixth decades of life [28]. The reported mean age is indicative of the late-onset nature of metastatic cancer, which typically arises after years of disease progression in many solid tumours. Age is a significant determinant of DnMC, with advanced age being consistently associated with an increased risk of metastatic spread at initial diagnosis [29, 30]. Elderly individuals often have more aggressive tumour biology and comorbidities, predisposing them to distant metastases [31].

The wide age range of 15–90 years, encapsulates the diverse age spectrum characteristic of patients diagnosed with DnMC. It reflects the reality that even though cancer is often a disease of aging, with genetic mutations, environmental exposures and lifestyle factors accumulating over time to increase cancer risk, metastatic cancer is not confined to only older populations. Younger patients (≤ 30 years) comprised 10.7% of the study participants and may have been diagnosed with more aggressive cancer subtypes, which tend to present at an advanced stage. Younger patients often present with cancers that have distinct biological characteristics, such as higher-grade tumours or hormone-receptor-negative breast cancers, which are known to have poorer prognoses and are more likely to present with metastases [32]. Younger patients with metastatic disease also face unique challenges in their cancer journey. Apart from dealing with the biological aggressiveness of their disease, younger individuals often encounter more significant psychosocial and emotional impacts due to the interruption of life milestones such as education, career development and family planning [33]. Moreover, younger patients may face delays in diagnosis due to the perception that cancer is primarily a disease of older adults, which can lead to misdiagnosis or the suboptimal evaluation of symptoms. This delay can be particularly pronounced in LMICs where healthcare infrastructure is limited, and cancer awareness is low.

In all, 28.8% of the participants reported a history of alcohol intake, whereas 13.7% had a history of smoking tobacco. Both alcohol consumption and the smoking of tobacco are established risk factors for various types of cancer including breast, liver and gastrointestinal cancers [34]. In the context of metastatic cancer, a history of alcohol use may contribute to the complexity of treatment and prognosis due to potential interactions with cancer therapies and increased risk of additional health complications. Tobacco smoking, reported in 13.7% of the patients, is similarly associated with an increased risk of cancer, particularly lung, H&N and bladder cancers [35, 36]. Smoking history in metastatic cancer patients can also complicate treatment regimens and exacerbate comorbid conditions, affecting overall patient outcomes [37]. Hypertensive heart disease was present in 26% of the study participants. Hypertension and its complications, including hypertensive heart disease, are significant comorbidities that can impact cancer treatment and outcomes. Patients with cardiovascular comorbidities often face increased risks of treatment-related complications, such as cardiotoxicity from certain chemotherapeutic agents [38]. Managing DnMC in the context of hypertensive heart disease requires careful coordination of oncological and cardiological care to mitigate adverse effects and optimise treatment efficacy. Diabetes mellitus, reported in 3.4% of the participants, is another relevant comorbidity. Diabetes has been linked to increased cancer risk and can also influence cancer progression and treatment response [39]. The presence of diabetes mellitus can complicate cancer management due to potential interactions with medications and the need for rigorous control of blood glucose levels. Furthermore, diabetes may exacerbate cancer-related symptoms and affect overall patient well-being [40]. The presence of comorbidities in older adults can further complicate the management of metastatic cancer, as these patients may be less able to tolerate aggressive treatments such as chemotherapy or surgery. This underscores the importance of individualised treatment plans that balance the aggressiveness of the disease with the overall health status of the patient, taking into consideration factors such as performance status, comorbidities and quality of life.

The BMI distribution provides critical insights into the nutritional status of cancer patients in the West African setting, particularly in the context of metastatic disease, where cachexia and weight loss are commonly observed. The relatively high proportion of patients within the healthy BMI range (51.6%) is somewhat unexpected given that all the patients had metastatic disease and were treatment-naïve. One plausible explanation is that many patients may have been diagnosed with DnMC relatively early in the metastatic process, before the development of significant cancer-related weight loss and cachexia. In some cases, patients with gastrointestinal cancers or other tumours that cause metabolic changes may experience weight loss more gradually, allowing them to maintain a normal BMI for a period before severe cachexia sets in [41]. Nevertheless, the proportion of underweight patients (20.8%) is concerning, as malnutrition is a common complication in cancer patients, particularly those with locoregionally advanced or metastatic disease. Cachexia in cancer patients is a multifactorial metabolic syndrome characterised by ongoing loss of skeletal muscle mass, which cannot be fully reversed by conventional nutritional support and leads to progressive functional impairment [42]. Cancer-related malnutrition and cachexia are associated with poor clinical outcomes, including reduced tolerance to treatment, lower quality of life and decreased survival rates [43]. The considerable proportion of underweight participants also reflects the socioeconomic realities of many cancer patients in LMICs. Limited access to food, financial constraints and the lack of comprehensive cancer care programs that include nutritional support contribute to the poor nutritional status of cancer patients in such settings [44]. The appropriate management of the nutritional status of cancer patients can enhance treatment efficacy and improve quality of life, especially for those receiving palliative care [41]. There is therefore, a need for comprehensive cancer care programs in West Africa that do not only focus on oncological treatment but also include nutritional interventions aimed at improving the overall health and resilience of patients.

The proportion of patients who were overweight (16.4%) and those in the obesity category (11.3%) highlight another dimension of metabolic challenges in cancer. Obesity is a well-established risk factor for various cancers, including breast [45], endometrial and colorectal cancers and is associated with a higher risk of developing more aggressive cancer types. Interestingly, patients who are overweight or obese may also have a paradoxically better prognosis compared to underweight patients due to a phenomenon known as the obesity paradox, wherein individuals with higher BMI may tolerate certain cancer treatments better or have more nutritional reserves to counteract the catabolic effects of cancer [46]. However, obesity presents challenges in cancer management, including difficulties in imaging, surgical complications and higher risks of comorbid conditions such as cardiovascular disease and diabetes, which can complicate cancer treatment [47]. The overall pattern of distribution of BMI categories reflects the dual burden of malnutrition and obesity, common in LMICs, where undernutrition coexists with the rising prevalence of obesity due to urbanization, dietary changes and lifestyle factors [48]. The presence of both extremes in the BMI spectrum highlights the need for tailored interventions that address both undernutrition and the health risks associated with obesity. Comprehensive cancer care for patients with DnMC must include nutritional assessments and individualised dietary interventions to optimise patient outcomes and improve treatment tolerability.

In all, 10.3% of the patients with DnMC reported a positive family history of cancer, which is in line with established data linking genetic predisposition to cancer risk. A positive family history of cancer is a well-known risk factor for several cancers, including breast, ovarian, colorectal and prostate cancers [49]. Identifying DnMC patients with a family history of cancer is crucial for guiding genetic counseling, testing and preventive strategies. In clinical practice, patients with a known family history of cancer may benefit from enhanced surveillance, preventive measures and potentially earlier intervention. In limited-resource settings, genetic counseling and testing are underutilised due to limited access to specialised healthcare services and lack of awareness. The low proportion of DnMC patients reporting a positive family history of cancer could be influenced by underreporting or unawareness of familial cancer risks [50].

The study identified pain as the most prevalent presenting symptom among patients with DnMC, in alignment with global research data indicating that pain is a common and significant symptom in metastatic cancer [51]. Pain management is crucial for maintaining quality of life and functional status, as metastatic disease often leads to pain due to tumour growth and bone metastases [52]. Bleeding, observed in 16.5% of the patients, was the second most frequent symptom that is often associated with malignancies involving mucosal surfaces or vascular tissues, such as gastrointestinal and gynecological cancers [53].

The study found that 68.2% of patients with DnMC had a good performance status (ECOG 0–1), while 14.7% had a poor performance status (ECOG 3). The ECOG performance status is a critical measure of a patient’s functional capacity and ability to undergo active treatment [54]. Some patients with DnMC may be diagnosed at an early stage of systemic involvement, where the primary tumour is more localised, and the metastatic burden is low. This can allow patients to maintain a higher functional status. The high proportion of patients with ECOG 0 –1 suggests that many patients were relatively well-functioning despite having DnMC. On the other hand, the presence of 14.7% of patients with ECOG 3 indicates considerable functional impairment. Such patients face significant challenges in managing their disease, often requiring enhanced supportive care and palliative interventions [55]. Identifying and comprehensively addressing the peculiar needs of DnMC patients with poor performance status are both essential for optimising care and improving quality of life.

Ultrasonography was the most frequently utilised imaging modality (60.3%) for diagnosing distant metastasis. Its high usage rate underscores its role in initial evaluations and subsequent monitoring, particularly for superficial metastases and guiding interventions. Ultrasonography’s non-invasive nature, cost-effectiveness and easy accessibility make it a valuable tool for evaluation of metastatic cancer, especially in settings where advanced imaging may be less available [56]. Overall, MRI and Technetium-99m bone scintigraphy had lower usage rates of 9.4% and 8.2%, respectively. The low utilization of MRI may be due to its higher cost and availability constraints, though it remains crucial for detailed soft tissue evaluation. In Ghana, there are only two centers capable of performing bone scans, and availability is limited; patients are typically scheduled in advance, with scans conducted periodically due to challenges associated with the cost and importation of the required radioactive material.

The prevalence of DnMC shows distinct patterns across various primary cancer sites and types. For example, colorectal cancer exhibits high DnMC prevalence rates of approximately 15%, possibly due to the aggressive nature of these tumours [57]. Conversely, patients with prostate cancer demonstrate lower rates of DnMC, ranging from 4% to 5% [58], aligning with the typically slower progression of localised prostate cancer to metastasis. Approximately, 4% to 10% of patients with nasopharyngeal carcinoma have distant metastasis at diagnosis [59]. However, the occurrence of DnMC at all sites of the H&N region accounted for 10.1% of the DnMC patients. The gastrointestinal tract was the commonest primary cancer site (25.8%) followed by the breast (17.6%) and the gynecological tract (17.0%). The kidney was part of the primary cancer sites among a small fraction of the patients (3.9%). However, renal cancers have been associated with significantly elevated prevalence rates of DnMC, ranging from 20% to 30% [60]. The relatively low rate observed in this study could be due to the generally low prevalence of renal cancers in Ghana [61]. The prevalence of breast cancer as a primary site of DnMC among the participants (17%) demonstrates its propensity to metastasise to distant sites. Breast cancer in young women with DnMC is considered a distinct biological entity. This theory is based on the observation that, even though mammography has led to the detection of more localised disease at diagnosis in women above >50 years, there has been no corresponding decline in cases of de novo metastatic breast cancer (DnMBC), suggesting that DnMBC could be a systemic disease that is already present at the time of initial diagnosis [62–64].

Additionally, DnMC patients of different ages have been shown to have distinct metastatic patterns and prognostic features. For example, de novo liver metastasis is more common among young breast cancer patients with better prognosis whereas lung metastasis occurs more frequently among older patients with poorer prognosis [65]. The most prevalent metastatic sites among the study participants were the lung (27.0%), liver (22.0%) and bone (9.4%). Generally, the prevalence of lung and liver metastases resonates with the commonality of these sites across various cancer types. The lung’s propensity to serve as a site for metastatic spread is due to its rich blood supply, while the liver’s role in filtering systemic blood circulation makes it susceptible to the dissemination of malignant cells. The occurrence of bone metastases emphasises the complex interaction between cancer cells and the bone microenvironment. The prevalence of specific histological types of DnMC can vary based on factors such as geographic location, patient demographics and advancements in detection and treatment modalities. Understanding the prevalence and characteristics of different histological types of DnMC is crucial for clinicians to tailor treatment strategies and improve patient outcomes. The study showed a significant proportion of adenocarcinomas (35.2%), aligning with the prevalence of primary cancer sites linked to this histology, such as the gastrointestinal tract, breast, gynecological system and the prostate gland [66].

EBRT was the most frequently utilised treatment modality, accounting for 26.7% of the treatments administered to patients with DnMC. It is a cornerstone in the management of metastatic cancer, particularly for palliating symptoms such as pain, bleeding and obstruction [67, 68]. Its role in palliative care for patients with metastatic disease is well-established [69]. The use of chemotherapy (25.8%) reflects its importance in managing systemic disease and addressing the metastatic spread, particularly in patients with a good performance status [55].

A significant proportion of patients with DnMC (86.7%) were managed with palliative intent, focusing on providing relief from symptoms and improving patients’ quality of life. Palliative care is essential in metastatic cancer, where the primary objective often shifts from curing the disease to managing symptoms, improving comfort and providing psychological and emotional support [70, 71]. Only 5% of patients were managed with curative intent. This low percentage underscores the challenging nature of DnMC, where curative treatment options are often limited due to the extent of disease spread [72]. Also, 8.3% received best supportive care alone, which involves managing symptoms and providing comfort without the use of disease-modifying therapies. This approach is typically reserved for patients with severe disease or those with poor performance status, too frail to tolerate active treatments [73–76].

## Recommendations

The results provide data in support of the urgent need to expand cancer awareness campaigns, optimise screening strategies and develop healthcare infrastructure to facilitate the early diagnosis and prompt treatment of cancer in West Africa. Future Research on DnMC in Ghana should be conducted over a much broader period to detect changes in the incidence of DnMC with time. Additionally, a broader sample including all the three major cancer treatment centres in Ghana should be adopted for future studies on DnMC. Further research is also needed to explore the long-term impact of the COVID-19 pandemic on cancer outcomes in this population.

## Limitations

The study relied on retrospective data, which was subject to incomplete medical records, missing information and variations in data accuracy over time. The study was conducted over a 2-year period and thus could not detect changes in the pattern of incidence of DnMC over time at the study site. Additionally, the study did not capture other DnMC patients who presented for treatment at peripheral healthcare facilities or regional hospitals. These factors might impact the overall representation of DnMC patients. Additionally, the COVID-19 pandemic coincided with the study period and may have affected the prevalence and clinicopathological characteristics of DnMC in the study population. Pandemic-related disruptions may have also delayed cancer diagnosis, contributing to a higher proportion of patients presenting with advanced disease. The exploration of these effects were beyond the scope of this study. Despite these limitations, the study provides valuable insights into the prevalence and clinicopathologic profiles of DnMC patients in a limited-resource setting.

## Conclusion

The study provides valuable insights into the landscape of DnMC in Ghana, shedding light on its prevalence, primary cancer sites and clinical characteristics. There was a relatively high rate of DnMC compared with high-income countries, emphasising the need for early detection and expanded access to comprehensive cancer care in limited-resource settings. The results underscore the critical need for focused research and tailored treatment strategies for this unique patient population.

## Conflicts of interest

The authors declare no competing interest.

## Funding

The study did not receive any specific funding support from funding agencies in the public, commercial or not-for-profit sectors.

## Author contributions

JD: Conceptualisation, Methodology, Validation, Supervision, Visualisation, Writing – Original Draft, Writing- Review & Editing OIA: Conceptualisation, Methodology, Formal analysis, Investigation, Writing – Original Draft AYN: Writing – Review & Editing EAA: Writing – Review & Editing JNOT: Writing – Review & Editing KAD: Writing – Review & Editing, Supervision.

## Data availability

The data used to support the findings of this study are available from the corresponding author upon reasonable request.

## Figures and Tables

**Table 1.** BMI of patients.

BMI group	BMI (kg/m^2^)	Frequency (*N* = 159)	Percentage (%)
Underweight	<18.5	33	20.8
Healthy range	18.5–24.9	82	51.6
Overweight	25–29.9	26	16.4
Obesity	30–39.9	18	11.2
Severe obesity	≥ 40	0	-

BMI: body mass index

**Table 2.** Imaging modalities used in the detection of distant metastatic cancer sites.

Imaging modality	Frequency	Percentage (%)
X-ray	72	45.2
Ultrasonography	96	60.3
CT scan	52	32.7
MRI	15	9.4
Technetium-99m bone scintigraphy	13	8.2

**Table 3.** Treatment intention for the patients with DnMC (*N* = 159).

Treatment intent	Frequency (*n*)	Percentage (%)
Curative treatment	8	5
Palliative treatment	138	86.7
Best supportive care Alone	13	8.3
